# Androgen receptor in advanced breast cancer: is it useful to predict the efficacy of anti-estrogen therapy?

**DOI:** 10.1186/s12885-018-4239-3

**Published:** 2018-03-27

**Authors:** Giuseppe Bronte, Andrea Rocca, Sara Ravaioli, Maurizio Puccetti, Maria Maddalena Tumedei, Emanuela Scarpi, Daniele Andreis, Roberta Maltoni, Samanta Sarti, Lorenzo Cecconetto, Anna Fedeli, Elisabetta Pietri, Valeria De Simone, Silvia Asioli, Dino Amadori, Sara Bravaccini

**Affiliations:** 1 Istituto Scientifico Romagnolo per lo Studio e la Cura dei Tumori (IRST) IRCCS, Via P. Maroncelli 40, 47014 Meldola, FC Italy; 2 S. Maria delle Croci Hospital, 48121 Ravenna, Italy; 3 Department of Pathology, Morgagni-Pierantoni Hospital, 47121 Forlì, Italy

**Keywords:** Androgen receptor, Advanced breast cancer, Anti-estrogen therapy, Endocrine therapy, AR/ER ratio

## Abstract

**Background:**

Androgen receptor (AR) is widely expressed in breast cancer (BC) but its role in estrogen receptor (ER)-positive tumors is still controversial. The AR/ER ratio has been reported to impact prognosis and response to antiestrogen endocrine therapy (ET).

**Methods:**

We assessed whether AR in primary tumors and/or matched metastases is a predictor of efficacy of first-line ET in advanced BC. Patients who had received first-line ET (2002–2011) were recruited, while those given concomitant chemotherapy or trastuzumab or pretreated with > 2 lines of chemotherapy were excluded. ER, progesterone receptor (PgR), Ki67 and AR expression were assessed by immunohistochemistry, and HER2 mainly by fluorescent in-situ hybridization. Cut-offs of 1 and 10% immunostained cells were used to categorize AR expression.

**Results:**

Among 102 evaluable patients, biomarkers were assessed in primary tumors in 70 cases and in metastases in 49, with 17 patients having both determinations. The overall concordance rate between primary tumors and metastases was 64.7% (95% CI 42%-87.4%) for AR status. AR status did not affect TTP significantly, whereas PgR and Ki67 status did. AR/PgR ≥0.96 was associated with a significantly shorter TTP (HR = 1.65, 95% CI 1.05-2.61, *p* = 0.028). AR status in primary tumors or metastases was not associated with progressive disease (PD) as best response. In contrast, Ki67 ≥ 20% and PgR < 10% showed a statistically significant association with PD as best response.

**Conclusions:**

AR expression does not appear to be useful to predict the efficacy of ET in advanced BC, whereas Ki67 and PgR exert a greater impact on its efficacy.

## Background

Androgen receptor (AR) is emerging as an important mediator in the biology and therapy of breast cancer (BC). It has a role in breast carcinogenesis, varying on the basis of the expression of other biomarkers, such as estrogen receptor (ER), progesterone receptor (PgR), human epidermal growth factor receptor 2 (HER2/neu), and a prognostic effect has also been observed [1].

AR is a nuclear transcription factor, which is activated by the binding of steroid hormone androgens, similarly to other members of this family of receptors including ER and PgR [2].

AR protein is expressed in about 70–90% of BC. Its levels may however vary depending on the location considered (cytoplasmic and/or nuclear), the cutoff of expression (≥1%, ≥10%), and the antibody used for immunohistochemistry (IHC) [3, 4].

AR expression in different BC subtypes is also variable. It is found in about 75% of ER-positive, 50–60% of HER2-enriched, and 20-40% of triple negative BC, respectively [5, 6].

Depending on the expression of other hormone receptor proteins and their ligands, the AR pathway may promote or inhibit cell proliferation. Moreover, this interplay between AR, ER and their ligands is complicated by the possible conversion of androgens to estrogens. A cross talk between AR pathway and HER2/neu pathway is also known [7, 8].

AR and ER can compete for the binding to estrogen response elements (EREs) on specific genes [9]. So the binding of AR to EREs reduces the estrogen proliferative action, thus inducing anti-proliferative effects. Conversely, ER can bind to androgen response elements (AREs), obtaining the opposite effect [10]. This mechanism could explain the role of AR in the resistance to standard endocrine treatments [11].

Moreover a high AR expression can activate the epidermal growth factor receptor (EGFR), promoting an agonist effect of tamoxifen on ER pathway, and this aberrant mechanism could be blocked by enzalutamide +/− gefitinib [12].

Currently AR is under investigation in the clinical setting as a therapeutic target in BC. Enzalutamide and other anti-androgens appear suitable treatment options for AR-expressing BC [13, 14].

A favorable prognostic role of AR in BC was identified in various studies. This effect was confirmed, in terms of both disease-free survival (DFS) and overall survival (OS), in a large meta-analysis including almost 8000 patients with stage I-III BC. In other studies AR expression independently predicted longer BC specific survival and lower risk of recurrence [5, 15, 16].

We previously showed the value of PgR and Ki67 to predict benefit from first-line endocrine therapy in a case series of patients with advanced luminal breast cancer [17]. In this study we explore, in the same case series, the prognostic/predictive effect of AR expression.

## Methods

### Study design

A retrospective analysis was performed for this study. Patients with locally advanced or metastatic BC histologically confirmed treated with first-line ET from 2002 to 2011 at Istituto Scientifico Romagnolo per lo Studio e la Cura dei Tumori, IRST (Meldola, Italy), were included. Patients who received concomitant chemotherapy or trastuzumab or pretreated with more than 2 lines of chemotherapy were excluded. All patients were aged ≥18 years and were followed up for a period ranging from 5 to 13 years. We collected data from medical records of these patients. Subsequently we gathered tumor tissue samples of these patients for AR assessment.

The original hematoxylin-eosin stained sections were reviewed by two pathologists responsible for selecting pathological inclusions representative of tumor tissue and for the analysis of clinical pathological features, such as histologic grade.

The predictive value of AR expression, alone or in relation to the other conventional biomarkers, to select the patients responsive to first-line endocrine therapy was the primary endpoint.

IRST and AVR (Area Vasta Romagna) Ethics Committee (approval no. 1164), reviewed and approved the study and informed consent was obtained from all patients.

Data were collected from electronic databases. The following characteristics were evaluated for each single patient.

Patient’s data: age at diagnosis. Tumor’s data: 1) histotype (ductal, lobular, other, unknown); 2) tumor classification at diagnosis (T1-4, unknown) according to the American Joint Committee on Cancer (AJCC) Cancer Staging Manual 7th Edition; 3) nodal classification (N0-3, unknown); 4) presence of metastases at diagnosis (M0-1); 5) ER, PgR, Ki67, HER2 status in primary tumor and metastasis. Treatments and outcomes: 1) adjuvant chemotherapy; 2) adjuvant ET; 3) type of first-line ET; 4) best objective response (complete response (CR), partial response (PR), stable disease (SD), PD, according to RECIST criteria); 5) time-to-progression (TTP), meant as the time in months from the beginning of first-line ET until progression or last tumor response evaluation available.

All datasets used and/or analyzed during the current study are available from the corresponding author on reasonable request.

### Biomarker detection

Hormone receptors, Ki67, HER2 and AR status of the primary tumor, metastasis or both were assessed for all patients enrolled. The major part (79%) of the biomarkers detection was performed in the Pathology Unit of Morgagni Pierantoni Hospital in Forlì [17].

Tumor samples obtained during surgery were fixed in neutral buffered formalin and embedded in paraffin. Four-micron sections were mounted on positive-charged slides for each patient (Bio Optica, Milan, Italy). REporting recommendations for tumour MARKer prognostic studies (REMARK) and the European Quality Assurance guidelines were used. Immunostaining for conventional biomarkers and AR expression was performed using the Ventana Benchmark XT staining system (Ventana Medical Systems, Tucson, AZ, USA) with the Optiview DAB Detection Kit (Ventana Medical Systems). ER, PgR, Ki67 (Leica, Novocastra, Newcastle, UK), HER2 (Dako, Carpinteria, CA, USA) and AR (SP107 Cell Marque, Ventana Medical Systems) antibodies were used. For ER, PgR, Ki67 and HER2 detection, tissue sections were incubated for 60 min with antibodies diluted 1:80, 1:40, 1:100 and 1:350, respectively, in antibody diluent (Ventana Medical Systems). The AR antibody was pre-diluted by the supplier. Sections were incubated for 16 min and automatically counterstained with hematoxylin II (Ventana Medical Systems). Biomarker positivity was detected and semiquantitatively quantified as the percentage between immunopositive tumor cells and the total number of tumor cells. Two independent observers evaluated all the samples and any disagreement was resolved by consensus after joint review using a multihead microscope. Hormone receptors, Ki67 and HER2 biomarkers were classified on the basis of the St. Gallen and ASCO-CAP guidelines [18–20]. In particular tumors were considered ER-positive when ≥1% of immunoreactive cells were detected. The cut-off for PgR positivity was set at ≥1% immunoreactive cells. Ki67 was defined as high when the fraction of positively stained cells was ≥20%, and low when < 20%. Fluorescent in situ hybridization, was used preferentially to determine HER2 status and considered positive if the HER2 gene to chromosome 17 centromere ratio was ≥2 or if the average HER2 gene copy number per cell was ≥6. In a minority of cases, HER2 was assessed using the HercepTest (DAKO Corporation) which measures the percentage of immunoreactive neoplastic cells defined according to the intensity and completeness of membrane staining and using the 0–3+ recommended scale. Cases scored as 3+ were considered HER2-positive. In cases of equivocal HER2 immunostaining (2+) FISH was performed [17]. As regards AR expression, tissues showing ≥1% or ≥ 10% positive tumor cells in their nucleus were defined as positive. The AR/ER and AR/PgR ratios were calculated as the ratio between the percentage of AR-positive tumor cells and the percentage of ER- or PgR-positive tumor cells, respectively.

### Statistical analysis

Patients and tumor features were described according to descriptive statistics.

Positive or negative AR status in both tumor and metastasis was used to define the concordance between tumor and metastasis, while discordance was defined as positivity at one site and negativity at the other one. The concordance rate was calculated as the proportion of concordant cases with respect to the total number of patients. The two-sided exact binomial 95% confidence interval (95% CI) was estimated.

AR expression was analyzed in relation to the other conventional biomarkers (ER, PgR, HER2 and Ki67), best response to therapy (CR, PR, SD, PD), and time to progression (TTP) (months). Kaplan-Meier method was used to estimate TTP and compared with the logrank test. Hazard ratios (HR) and their 95% CI were calculated using the Cox regression model.

The Chi Square test was used to evaluate the association between categorical variables and best response and the non-parametric ranking test (Wilcoxon test) was used to evaluate the relationship between median value of AR expression and Ki67 classes (< 20%, ≥20%).

To determine the optimal cutoff values of AR/ER and AR/PgR ratios at a median TTP time Receiver operating characteristic (ROC) curves were used.

Analyses were performed using SAS Statistical software (version 9.4, SAS Institute, Cary, North Carolina, USA). Statistical tests were significant for *p* values < 0.05.

## Results

### Patients’ characteristics

One hundred and two patients were evaluable according to the selection criteria and the availability of tissue samples. Median age was 60 years (range 33-85). Seventy eight percent of them were diagnosed as ductal and 14% as lobular histotype. Metastatic disease at diagnosis was found in 26 out of 102 (25.5%). Ninety two percent were treated with an aromatase inhibitor as first-line endocrine therapy, and letrozole was the most frequent aromatase inhibitor administered (45%). For detailed description of patients’ characteristics see Table 1.

**Table 1:** Patient’s characteristics

No. patients	102 (100)
Age (years): median value (range)	60 (33-85)
Previous adjuvant chemotherapy	
No	37 (36.3)
Yes	39 (38.2)
Unknown/Not available	26 (25.5)
Previous adjuvant endocrine therapy	
No	22 (21.6)
Yes	54 (52.9)
Unknown/Not available	26 (25.5)
Histotype	
Ductal	76 (74.5)
Lobular	14 (13.7)
Other	7 (6.9)
Unknown	5 (4.9)
Tumor stage	
1	30 (29.4)
2	42 (41.1)
3	2 (2)
4	12 (11.8)
Unknown	16 (15.7)
Grade of primary tumor	
1	2 (2)
2	34 (33.3)
3	32 (31.4)
Unknown	34 (33.3)
Nodal involvement	
0	24 (23.5)
1	36 (35.3)
2	11 (10.8)
3	11 (10.8)
Unknown	20 (19.6)
Metastases (at diagnosis)	
0	76 (74.5)
1	26 (25.5)
Type of first-line endocrine therapy	
Letrozole	46 (45.1)
Anastrozole	22 (21.6)
Exemestane	26 (25.5)
Tamoxifen	5 (4.9)
Fulvestrant	3 (2.9)

### Biomarkers’ distribution

**Fig. 1:**
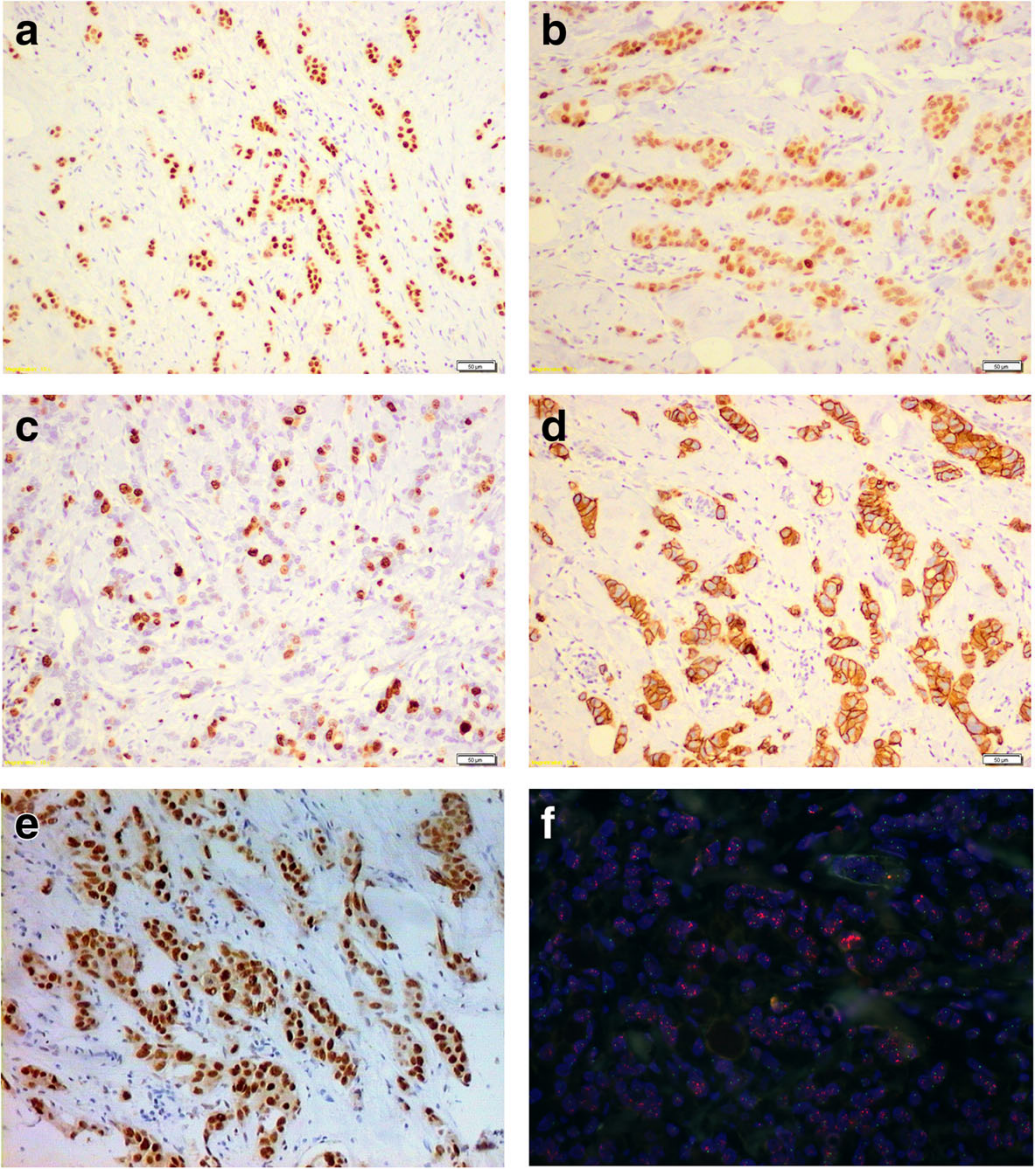
Biomarkers detection in breast cancer tissue. **a** ER expression by immunohistochemistry (10X magnification). **b** PgR expression by immunohistochemistry (10X magnification). **c** Ki67 expression by immunohistochemistry (10X magnification). **d** HER2 expression by immunohistochemistry (10X magnification). **e** AR expression by immunohistochemistry (10X magnification). **f** HER2 amplified case by Fluorescence in situ hybridization (FISH) (40X magnification)

Biomarker’s expression (ER, PgR, Ki67, HER2 and AR) was assessed in primary tumors in 70 cases and in metastases in 49, with 17 patients having both assessments (Fig. 1a-f) (Table 2).

**Table 2:** Biomarkers determination

	Primary tumor (*n* = 70)	Metastases (*n* = 49)	Clinical practice^a^ (*n* = 102)
	No. (%)	No. (%)	No. (%)
ER status			
< 1%	3 (4.3)	0	2 (2.0)
≥ 1%	65 (92.9)	49 (100)	100 (98.0)
Unknown	2 (2.8)	0	0
PgR status			
< 1%	10 (14.3)	12 (24.5)	22 (21.6)
≥ 1%	58 (82.9)	37 (75.5)	80 (78.4)
Unknown	2 (2.8)	0	0
Ki67 status			
< 20%	39 (55.7)	30 (61.2)	63 (61.8)
≥ 20%	27 (38.6)	17 (34.7)	38 (37.2)
Unknown	4 (5.7)	2 (4.1)	1 (1)
HER2 status			
Negative	57 (81.4)	41 (83.7)	88 (86.3)
Positive	10 (14.3)	7 (14.3)	14 (13.7)
Unknown	3 (4.3)	1 (2)	0
AR status			
< 1%	5 (7.1)	12 (24.5)	17 (16.7)
≥ 1%	65 (92.9)	37 (75.5)	85 (83.3)
< 10%	7 (10.0)	19 (38.8)	26 (25.5)
≥ 10%	63 (90.0)	30 (61.2)	76 (74.5)

*Abbreviation*: *ER* estrogen receptor, *PgR* progesterone receptor, *AR* androgen receptor, *HER2* human epidermal growth factor receptor 2

^a^biomarker measured on metastatic sample when a metastatic biopsy was available, or on primary tumor when biopsy of metastasis had not been performed

ER was negative (< 1%) in 3 samples (4.3%) of primary tumors and in none of the metastatic samples. PgR was negative (< 1%) in 10 (14.3%) primary tumors and in 12 (24.5%) metastases. Ki67 was low (< 20%) in 59.1% of primary tumors and in 61.2% of metastases. HER2 status was positive in about 15% of cases both in primary tumors and metastases. In Fig. 1f, a HER2 FISH amplified case has been reported.

**Fig. 2:**
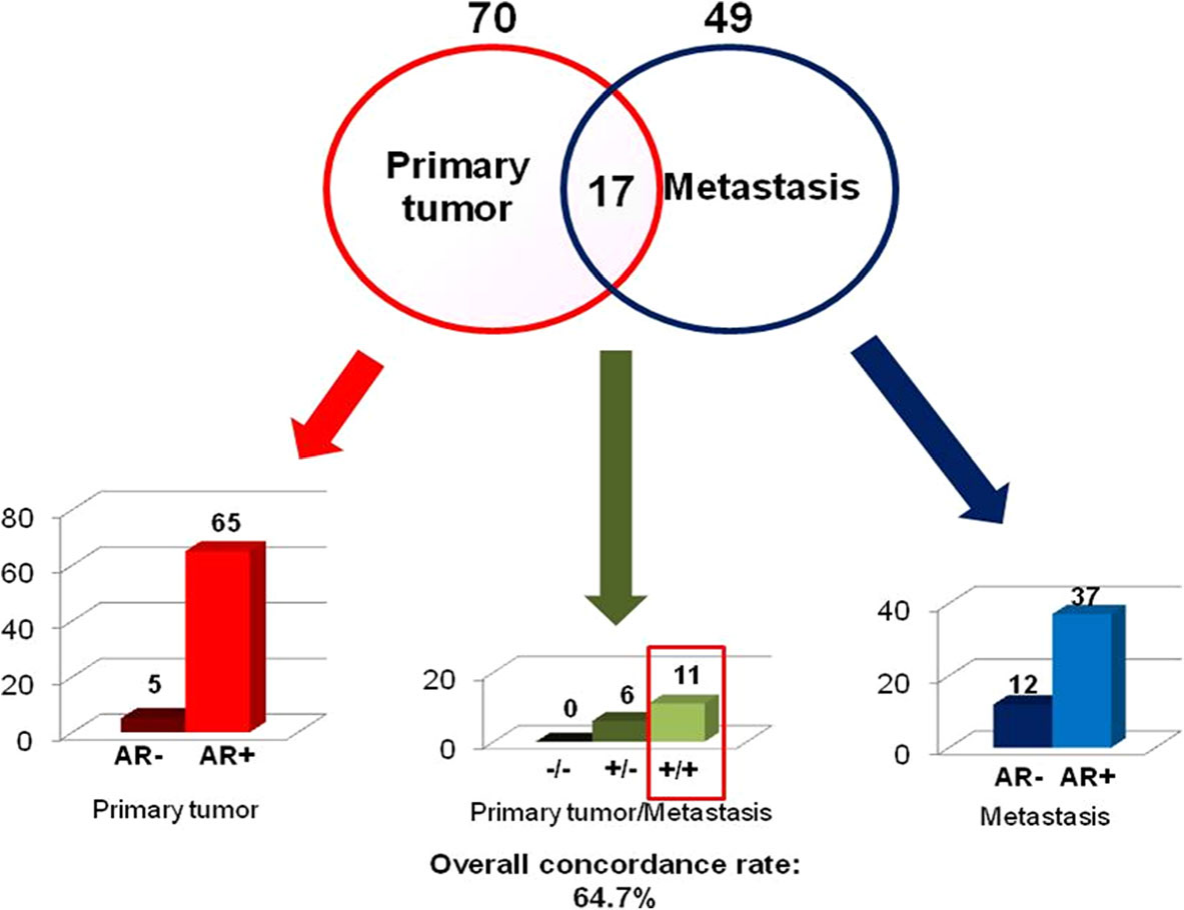
Distribution of AR expression in primary tumor and metastasis and concordance

AR status (Fig. 1e), considered as per clinical practice (biomarker measured on a metastatic sample when a metastatic biopsy is available, or measured on primary tumor when biopsy of a metastasis has not been performed), was negative in 17 (16.7%) cases with cutoff < 1% and 26 (25.5%) cases with cutoff < 10%. The overall concordance rate between primary tumors and metastases was 64.7% (95% CI 42.0%–87.4%) for AR expression, based on cutoff of 1% (Fig. 2). Furthermore we observed a statistically significant association of AR status with a low Ki67, with median value of AR expression as per clinical practice of 80% in patients with Ki67 < 20% versus 70% in patients with ki67 ≥ 20% (*p* = 0.017).

### Outcome measures

AR status in primary tumors or metastases was not associated with PD as best response (Table 3).

**Table 3:** Response to treatment in relation to biomarkers expression considered as per clinical practice^a^

	No.	CR or PR or SD	PD	*p*
		No. (%)	No. (%)	
ER status				
< 1%	1	0	1 (100)	
≥ 1%	72	64 (88.9)	8 (11.1)	0.123
PgR status				
< 1%	15	11 (73.3)	4 (26.7)	
≥ 1%	58	53 (91.4)	5 (8.6)	0.079
Ki67 status				
< 20%	46	44 (95.7)	2 (4.3)	
≥ 20%	26	19 (73.1)	7 (26.9)	0.009
HER2 status				
Negative	64	57 (89.1)	7 (10.9)	
Positive	9	7 (77.8)	2 (22.2)	0.306
AR status				
< 1%	12	10 (83.3)	2 (16.7)	
≥ 1%	61	54 (88.5)	7 (11.5)	0.619
< 10%	19	15 (79.0)	4 (21.0)	
≥ 10%	54	49 (90.7)	5 (9.3)	0.182
AR/ER ratio				
< 0.90 (best cutoff)	38	31 (81.6)	7 (18.4)	
≥ 0.90 (best cutoff)	35	33 (94.3)	2 (5.7)	0.101
AR/PgR ratio				
< 0.96 (best cutoff)	27	23 (85.2)	4 (14.8)	
≥ 0.96 (best cutoff)	46	41 (89.1)	5 (10.9)	0.623

*Abbreviation*s: *CR* complete response, *PR* partial response, *SD* stable disease, *PD* progressive disease, *ER* estrogen receptor, *PgR* progesterone receptor, *AR* androgen receptor, *HER2* human epidermal growth factor receptor 2

^a^biomarker measured on metastatic sample when a metastatic biopsy was available, or on primary tumor when biopsy of metastasis had not been performed

Clinical benefit rate, defined as CR or PR or SD, was 88.5% and PD 11.5% for AR-positive tumors defined by a cutoff ≥1%, versus 83.3% and 16.7%, respectively, for AR-negative tumors (*p* = 0.62); clinical benefit was 90.7% and PD 9.3% for AR-positive tumors defined by a cutoff ≥10%, versus 79% and 21%, respectively, for AR-negative tumors (*p* = 0.18). Conversely Ki67 showed a significant association with PD as best response, with 26.9% PD with high Ki67 versus 4.3% PD with low Ki67 (*p* = 0.009). PgR-negative status defined by a cutoff < 1% showed just a trend in terms of association with PD as best response (*p* = 0.079), while significant results (*p* = 0.031) were obtained by using ≤10% PgR as cut off value.

**Fig. 3:**
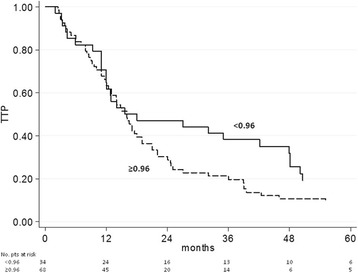
TTP as a function of AR/PgR ratio

Median TTP was 17 months (95% CI 14-21.5, median follow-up 75 months). Differences in TTP according to AR status were not statistically significant (Table 4). For AR expression ≥1% median TTP was 16.1 months (95% CI 13.0-19.0), versus 12 months (95% CI 4.3-48.1) for AR < 1% (*p* = 0.884); similarly for AR ≥10% median TTP was 16 months (95% CI 13.0–19.0) vs 13.8 months (95% CI 11.0–42.1) for AR < 10% (*p* = 0.935). AR/PgR ≥0.96 was associated with a significantly shorter TTP (HR = 1.65, 95% CI 1.05-2.61, *p* = 0.028) (Fig. 3). No associationwas found between AR/ER ratio and TTP. Conversely, a positive PgR status and a low Ki67 (but not HER2 status) were significantly associated with longer TTP (Table 4).

**Table 4:** Time to progression (TTP) according to biomarkers expression considered as per clinical practice^a^

	No.	HR (95% CI)^b^	Median TTP, months (95% CI)	p
AR status				
< 1%	17	1.00	12.0 (4.3-48.1)	
≥ 1%	85	0.96 (0.56-1.66)	16.1 (13.0-19.0)	0.884
< 10%	26	1.00	13.8 (11.0-42.1)	
≥ 10%	76	0.98 (0.61-1.57)	16.0 (13.0-19.0)	0.935
AR/ER ratio				
< 0.90 (best cutoff)	52	1.00	12.9 (11.0-17.1)	
≥ 0.90 (best cutoff)	50	0.83 (0.55-1.24)	18.0 (14.0-24.6)	0.362
AR/PgR ratio				
< 0.96 (best cutoff)	34	1.00	16.8 (12.1-47.9)	
≥ 0.96 (best cutoff)	68	1.65 (1.05-2.61)	16.0 (12.4-19.0)	0.028
PgR status				
< 1%	22	1.00	10.5 (4.0-17.0)	
≥ 1%	80	2.18 (1.34-3.55)	17.0 (14.0-24.7)	0.001
Ki67 status				
< 20%	63	1.00	17.6 (14.8-22.1)	
≥ 20%	38	1.60 (1.05-2.45)	12.0 (8.2-16.1)	0.028
HER2 status				
negative	88	1.00	15.8 (12.9-19.0)	
positive	14	1.15 (0.65-2.02)	18.0 (7.0-46.0)	0.929

*Abbreviations*: *ER* estrogen receptor, *PgR* progesterone receptor, *AR* androgen receptor, *HER2* human epidermal growth factor receptor 2

^a^biomarker measured on metastatic sample when a metastatic biopsy was available, or on primary tumor when biopsy of metastasis had not been performed

^b^HR hazard ratio

## Discussion

Most of ER-positive BCs are AR-positive (about 80-90% of them) and the coexpression of AR, ER and PgR is associated with a better prognosis and well-differentiated phenotype [21–23].

Some studies suggest that the levels of expression of AR and its relation to ER expression levels in primary tumors predict benefit from adjuvant endocrine therapy with tamoxifen [11].

This study was not powered to determine whether AR expression could predict response to tamoxifen or fulvestrant, as the majority of patients received aromatase inhibitors and very few patients received those therapies.

Nevertheless our findings suggest that AR expression does not predict the efficacy of first-line endocrine treatment (mainly based on aromatase inhibitors in our study) in ER- or PgR-positive advanced BC, both in term of TTP and PD as best response.

These results might be influenced by the limited amount of AR negative cases in this subtype of BC. Moreover, the stronger predictive effect of PgR and Ki67 could make the role of AR expression less evident, as suggested by the predictive significance of the AR/PgR ratio.

Cochrane et al. [11]. evaluated nuclear protein expression levels of AR and ER because previous studies reported that AR mRNA and protein decrease in tumors responsive to neoadjuvant endocrine therapy [24, 25]. The best cutoff point for AR/ER ratio was 2.0 as determined by ROC analysis in the study by Cochrane et al. We evaluated, for the first time, both AR/ER ratio and AR/PgR ratio in a subset of metastatic BC patients, to assess their predictive potential for efficacy of endocrine therapy. These ratios were measured as the percentages of tumor positive cells for each receptor, through immunohistochemical staining. In our study, the ROC analysis identified 0.9 as the best cut-off value for AR/ER ratio, which differed from the one calculated by Cochrane et al. probably due to the different subset of patients analyzed, and was not associated with outcome.

Moreover we evaluated AR/PgR ratio and we found a potential predictive value of this parameter at the cut-off of 0.96. This finding could be explained by the stronger predictive value of PgR alone in comparison with AR alone, as PgR < 10% and Ki67 ≥ 20% showed a significant association with PD as best response, and PgR ≤1% and Ki67 ≥ 20% were significantly associated with shorter TTP.

## Conclusions

PgR and Ki67 seem to be useful to select patients with a higher probability of being responsive to first-line endocrine therapy for metastatic BC, whereas AR expression does not appear to be useful. The AR expression could acquire more relevance when anti-androgen therapy will be available for BC patients.

## Abbreviations

AR: Androgen receptor
ARE: Androgen response element
BC: Breast cancer
CR: Complete response
DFS: Disease-free survival
EGFR: Epidermal growth factor receptor
ER: Estrogen receptor
ERE: Estrogen response element
ET: Endocrine therapy
HER2/neu: Human epidermal growth factor receptor 2
HR: Hazard ratio
IHC: Immunohistochemistry
OS: Overall survival
PD: Progressive disease
PgR: Progesterone receptor
PR: Partial response
ROC: Receiver operating characteristic
SD: Stable disease
TTP: Time to progression
